# Cognitive Bias in Zoo Animals: An Optimistic Outlook for Welfare Assessment

**DOI:** 10.3390/ani8070104

**Published:** 2018-06-27

**Authors:** Isabella L. K. Clegg

**Affiliations:** Animal Welfare Expertise (www.animalwelfareexpertise.com), 81 Alderney St, London SW1V 4HF, UK; izziclegg@hotmail.co.uk

**Keywords:** affective state, animal-based measures, animal welfare, cognitive bias, zoo animals

## Abstract

**Simple Summary:**

Cognitive bias testing has emerged as one of the most valid tools in measuring animals’ affective states, and while it has been extensively applied in farm and laboratory settings, only a few studies have taken place in zoos and aquaria. This review evaluates past cognitive bias studies on non-domesticated, “exotic” species kept in zoos or other settings and uses their experiences to make recommendations for establishing this research in zoos. The many variables inherent to functioning zoo environments will determine the scope and design of cognitive bias studies, but equally future efforts should be cognizant of the significant and unique benefits for the animals, managers, and scientists involved.

**Abstract:**

Cognitive bias testing measures how emotional states can affect cognitive processes, often described using the “glass half-full/half-empty” paradigm. Classical or operant conditioning is used to measure responses to ambiguous cues, and it has been reported across many species and contexts that an animal’s cognitive bias can be directly linked to welfare state, e.g., those in better welfare make more optimistic judgements. Cognitive bias testing has only recently been applied to animals and represents a key milestone in welfare science: it is currently one of the only accurate methods available to measure welfare. The tests have been conducted on many farm, laboratory, and companion animal species, but have only been carried out in zoo settings a handful of times. The aims of this review are to evaluate the feasibility of cognitive bias testing in zoos and its potential as a tool for studying zoo animal welfare. The few existing zoo cognitive bias studies are reviewed, as well as those conducted on similar, non-domesticated species. This work is then used to discuss how tests could be successfully designed and executed in zoo settings, which types of tests are most appropriate in different contexts, and how the data could be used to improve animal welfare. The review closely examines the many variables are present in the zoo which cannot be controlled as in other settings, termed here the Zoo Environment (ZE) Variables. It is recommended that tests are developed after consideration of each of the ZE Variables, and through strong collaboration between zookeepers, managers, and academic institutions. There is much unexplored potential of cognitive bias testing in the zoo setting, not least its use in investigating animal welfare in zoos. It is hoped that this review will stimulate increased interest in this topic from zoo managers, scientists, and industry regulators alike.

## 1. Introduction

The cognition-emotion interface is a two-way relationship: just as cognition may influence how we develop and experience emotions, emotional state can affect how we process information [1]. The latter interaction can be described using the umbrella term “cognitive bias”, and is demonstrated by examples such as people with depression making more negative judgements about an ambiguous event than non-depressed people [2,3]. Tests of cognitive bias can therefore be used to assess an individual’s emotional state, and for this reason, their application to animal models for the first time in the early 2000s [4] was ground-breaking for the fields of animal emotion and welfare.

Since the genesis of animal welfare science in the 1960s, researchers have been striving to find accurate and reliable measures of welfare for several species. Although there are still on-going discussions about its definition, animal welfare is generally defined as “feelings-based” [5,6] and a practical definition in this vein by [7] (p. 159) describes it as the balance of positive and negative affective states. Affective states, and the shorter-term emotions they are made up of [8], typically have measurable components in the behavioral, physiological and cognitive categories [9,10]. Animal welfare scientists therefore aim to apply multiple measures in these categories at the same time to evaluate overall welfare [8,11]. Cognitive bias testing is the first tool to offer a non-invasive, conditioning method to assess how animals’ emotions affect their cognition, and has been supported by results from many species supporting construct and external validity [1].

The number of cognitive bias studies on farm, laboratory and companion animals has grown exponentially since the first examples, with the majority using judgement bias tasks to demonstrate that animals in poorer welfare conditions will judge ambiguous stimuli more pessimistically, and vice versa (see reviews by [1,12,13]). Despite this progress, only three cognitive bias studies have been conducted on animals kept in zoo settings: on bottlenose dolphins (*Tursiops truncatus*) [14], western lowland gorillas (*Gorilla gorilla gorilla*) [15] and an American black bear (*Ursus americanus*) [16]. Zoos and aquaria are facing increasing pressure from the public to monitor and improve animal welfare [17,18], alongside calls for scientists in the field to be attached to such institutions to aid with research and increase objectivity [19]. Cognitive bias testing has the potential to provide objective information on zoo animal welfare and is methodologically feasible for zoo species, but has not been widely applied in this setting [14,20,21]. There have been numerous past reviews of cognitive bias studies in animals, regarding the test methodology [12,21], the results’ accuracy in indicating emotional state [13], and the statistical approaches taken to interpret results [22]. Therefore this review will not be revisiting these topics in detail (readers are encouraged to look through this material to put the current work into context), and instead aims to highlight the potential of cognitive bias tests on species in zoos and aquaria (hereafter zoos), compile past cognitive bias studies on zoo-housed species and non-domesticated, “exotic”, species kept in other settings, and provide recommendations for how to effectively design and execute the tests given the unique factors of this setting.

## 2. Cognitive Bias and Animal Welfare

Originating from human psychology, patients reporting negatively-valenced affective states, such as anxiety or depression, were found to perform significantly differently in cognitive bias tests to people in more positive states [1,23]. There are several types of cognitive bias, which are measured differently, but in general people experiencing negative affect will make more pessimistic judgements about ambiguous stimuli (judgement bias), pay more attention to negative stimuli (attention bias), and remember negatively-valenced memories more readily (memory bias) than those in more positive affective states [2,3,23]. Judgement bias tests are by far the most common type applied to animals when measuring cognitive bias, perhaps because the tasks are more easily defined and controlled relative to the other types of bias. These tests have shown that animals in stressful, fear-inducing conditions, in a barren environment, or where negative affect is pharmaceutically induced, will make more pessimistic judgements [1,12,13]. Although less investigated, the same is true regarding positive affect, where for example more optimistic biases were shown in: domestic piglets (*Sus scrofa domesticus*) after gentle handling [24], laying chicks (*Gallus gallus domesticus*) in a more complex environment [25], and in numerous species when environmental enrichment was provided (see Table 1 of Supplementary Materials in [13]).

Animal welfare is described as the balance of positive and negative affective states [7]: an all-encompassing and subjective concept which is therefore difficult to measure. Finding valid markers of the behavioral, physiological and cognitive elements of emotions, and then considering them together to get a better picture of overall welfare, is a common approach to welfare assessment [10,11,26]. Behavioral and physiological indicators have been defined for several different species using a range of techniques, such as putting animals in certain contexts, spontaneous observations, and pharmaceutical interventions [27,28,29]. Initially the focus was on measuring negative emotions e.g., pain, fear, anxiety, since these were easier to identify, but more recently it has been highlighted that recognizing and promoting positive emotions is crucial to ensuring good welfare [10,30]. Although determining cognitive indicators of emotion is more difficult, they offer substantial benefits to emotion measurement such as the ability to reflect both the valence and arousal of an emotion, whereas behavioral and physiological indicators are generally accurate for only one of these axes [1,23]. This is especially true for zoo animal welfare indicators, where existing behavioral and physiological measures tend to focus on measuring arousal levels and are often very species-specific [31,32,33,34]. Cognitive bias results offer information on overall affective state and can distinguish between high or low arousal and valence [1,8,35], and would therefore lead to more meaningful welfare information and management decisions in zoos. As in [1] (p. 163), “cognitive” here describes different information processing functions including attention, learning, memory and decision-making. In the past, cognitive indicators of emotion were predominantly investigated using invasive methods, and thus were less relevant for real-time welfare assessment, or were limited by the fact that the equivalent methods in humans involved language-based tasks and thus could not be adapted for animals [23]. Therefore the discovery and application of cognitive bias tests on animals had significant implications for the field of welfare science, providing a non-invasive and comprehensive measure of affective state which could be adapted across species [1,21]. These past studies on laboratory, companion and farm animal species have used different ways to design and execute cognitive bias tests. To consider which protocols might be most applicable for conducting this research on zoo animals, the different methodological elements of the tests and their functions are outlined below.

## 3. Measuring Cognitive Bias

In this section, the focus will remain on measuring judgement biases, but memory and attention bias tests will also be described briefly. The principle of judgement bias tests is this: the subject learns that two distinct cues result in two different outcomes (usually one positive and one negative), and when presented with cues whose discriminative stimuli fall in-between these “conditioned cues”, makes an observable judgement about what will result from these “ambiguous” or “probe” cues, which are generally non-rewarded or only sporadically rewarded [13,36]. For example, in [37] (pp. 162–164) the dog subject is presented with either a full bowl on one side of the room, or an empty bowl on the other side. After repeated trials the dog is running faster to the side where the bowl is always full, and slower to the position where it is empty. This difference in speed signals that the dog has learnt to discriminate between the conditioned cues, and “ambiguous cue” trials are then conducted where the bowl is presented at the position directly in the middle of the positive and negative positions, and at locations slightly to the left and right (all equidistant from the start position). The dog’s speed to run to these bowls, either relatively fast (optimistic) or slow (pessimistic), is a measure of it is willingness to take risks and therefore used as the measure of bias. Studies involving judgement bias tasks follow this model but the test design itself often varies, for example by using either spatial, visual, auditory or olfactory dimensions for the differentiation of cues [13]. In general the responses to cues are part of either Go/No-Go or Go/Go tasks: in the former the animal must actively respond to the positive conditioned cue and suppress its response for the negative cue, whereas in Go/Go tasks active, but differing, responses are required for both conditioned cues [13].

To link judgement bias to welfare, the most common approach is for investigators to expose subjects to an emotion-inducing condition, such as barren versus enriched housing, using a within- or between-subjects design [13,21]. A more recent but less common approach is based on the assertion that individual differences in personality and mood might significantly affect cognitive bias [12,38,39]: instead of inducing a purported “good” or “poor” welfare situation on subjects, their cognitive biases have been linked to differences in personality [40,41], spontaneous behavior linked to affective state [14,42,43], and possibly moods induced by the tests themselves [44]. Other, less fundamental methodological variables include the nature of the reinforcement/punishment, how the subjects are conditioned to the cues, the criteria needed to pass to the testing phase, the conditions the subject faces during testing (e.g., socially isolated or not), and the total number of trials (see [13] (pp. 4–10) for discussions on these). Attention and memory biases have been also been investigated in animals but in contrast to judgement bias studies, this research has been carried out predominantly in laboratory settings (mostly on rodents), and often with the aim of developing models of human psychopathology [45]. For example, positive or negative emotional states are induced in the subject and then the attention given to threatening or neutral stimuli is measured [45,46], or the recall ability of unpleasant memories evaluated [47,48]. However, more recently these biases are being investigated in terms of what they tell us about animal emotions and welfare [45], with concurrent efforts focusing on developing more practical test methodologies [49]. Lastly, an approach based on the ambiguous-cue paradigm (ACP) has been adapted to test cognitive bias in animals [15,16]. The ACP is used to study learning mechanisms in humans and non-human animals. It differs from spatial or other types of judgement bias task in that intermediate stimuli are not presented along the same dimension as the learned cues, in order to prevent responses simply reflecting “a perceptual discrimination of stimuli closer to reward and non-reward contingencies” [16]. Instead, the ambiguous stimuli are paired with novel cues, where the subject choosing the ambiguous cue over the novel cue reflects an optimistic decision and vice versa [15,16].

## 4. Cognitive Bias Testing on Exotic Species Housed in Zoos and Other Settings

In this section the few cognitive bias studies conducted in zoos are reviewed in terms of their feasibility and validity, and are then compared to bias studies on species commonly kept in zoos but carried out in other settings (Table 1).

### 4.1. Cognitive Bias Tests in Zoos

The three studies carried out within the zoo environment were all published in the same year but used different paradigms to test judgement bias. Researchers at Parc Astérix, France, tested eight bottlenose dolphins’ judgement biases in a spatial location task, and found that dolphins who judged more optimistically were also those who spent more time synchronously swimming with partners [14]. There were several noteworthy elements of this study that were different to most of the previous judgement bias studies: the animals were not socially isolated and thus tested in the presence of conspecifics; there was no “negative” learned cue (e.g., punishment) but instead a “le ss-positive” cue (compared to the positive cue); and the animals were not experimentally exposed to a welfare-inducing condition, where instead bias results were correlated with certain spontaneous behaviors. These conditions were essential to conduct the test in this setting: the dolphins would have likely been stressed by social isolation [56] as this did not occur often at the facility, and any experimental manipulation aiming to manipulate the animals’ welfare or using methods other than positive reinforcement training (PRT) would not have been acceptable to the management team. The authors published a second paper from this data where they looked further at the results from each ambiguous cue and found that the dolphins’ anticipatory behavior before food-provision training sessions was also correlated with their cognitive bias [36]. The more pessimistic dolphins showed higher levels of anticipatory behavior, in agreement with the reward-sensitivity paradigm where animals in poorer welfare will anticipate their rewards more, since there are fewer of such positive events in their environment [57,58]. These two zoo cognitive bias results highlighted the first potential welfare indicators in dolphins, both of which have direct implications for how the animals are housed and managed [59]. For example, as a result the dolphins’ caretakers were able to monitor each animal’s anticipatory behavior before training sessions and make an informal judgement about how stimulated it was by its environment and social group at that time, subsequently providing more environmental enrichment if necessary (I. Clegg, personal observation, June 2017).

The other two cognitive bias studies conducted in the zoo setting were the result of a collaboration between Oakland University and Detroit Zoological Society (Detroit, MI, USA), where three western lowland gorillas and an American black bear were tested using the ambiguous-cue paradigm (ACP) [15,16]. Overall, the subjects of these two studies had difficulties learning the pairings required for the ACP, and therefore judgement biases were not able to be tested, nor their correlation to the two chosen conditions (provision of forage enrichment and increased visitor density). However the authors argued that the differential rate of learning for the positive and negative valence cues, which varied between the three gorillas, was likely an indicator of affective state in itself and therefore merited further investigation [16].

### 4.2. Cognitive Bias Tests on Zoo Species Kept in Other Settings

Previous to the first cognitive bias tests being conducted in functioning zoos, several studies had been carried out in laboratories or research centers on some species that are often kept in zoos (i.e., non-domesticated, ‘exotic’ animals [62]). For example, regarding judgement bias investigations, a Go/No-Go task were successfully taught to rhesus macaques (*Macaca mulatta*) and the authors found that subjects judged more pessimistically after veterinary examinations and more optimistically after enrichment provision [51]. Attention bias has also been tested in rhesus macaques by this research group, where undergoing a veterinary examination significantly mediated the subjects’ attention towards an image of an aggressive conspecific [45]. When common marmosets (*Callithrix jacchus*) were tested, left-handed animals showed more pessimistic responses in a judgement bias task [54], while another study found no correlation between bias and rearing conditions [50]. White-lipped peccaries (*Tayassu pecari*) showed more pessimistic judgements after being net-trapped [58], and collared peccaries (*Pecari tajacu*) were more pessimistic in response to space restrictions in their environment [59]. Grizzly bears (*Ursos actos horribilis*) did not show any significant judgement bias differences after accessing various types of enrichment [55] in an experiment which only used differing levels of positive reinforcement. Another judgement bias study only using positive reinforcement found that more dominant tufted capuchins (*Cebus apella*), as well as those who received more grooming from conspecifics, judged more optimistically [61]. In a unique study which correlated behavioral, physiological and cognitive measures (the most accurate way to measure welfare [8]) also on tufted capuchins, it was found that animals performing more stereotypic “head-twirls” also had higher fecal corticoid levels and made more pessimistic judgements [60]. There has been one cognitive bias study carried out in a wildlife rescue center, aiming to develop a test using “cheap, low-tech” equipment to encourage future application in this setting [36]: the authors developed a simple task for chimpanzees (*Pan troglodytes*) and found individual differences in judgement bias, which they hypothesized may be due to social hierarchy positions (data taken in an earlier study).

Although not a species commonly kept in zoos, wild-caught European starlings (*Sturnus vulgaris*) have been the subject of a few judgement bias studies which may be applicable to designing tests for other bird species are kept zoos. Larger, enriched cages induced optimism in these birds [63,64], while those performing more stereotypic behavior made more pessimistic choices [65]. This research group also investigated the possibility of testing cognitive bias using naturally aversive stimuli to wild-caught starlings, where no associative training was needed [66]: unfortunately the birds’ cognitive bias was not predicted by the aversive condition (predator call playback), but this experimental model may be of interest to zoo researchers designing minimally invasive studies or working with untrained animals.

Lastly, a new model for testing cognitive bias has recently been proposed following studies on rhesus macaques, again resulting from the desire to reduce the time spent and complexity of training for the test. In addition to judgement, attention and memory biases, it has been shown that ‘response-slowing’ to cognitive tasks is also ‘biased’ by emotional state [52]. Response-slowing describes the impairment to cognitive performance dependent on affective state, usually experimentally induced by presenting either positive or negative ‘emotional distractor content’. Rhesus macaques that had undergone a veterinary examination were slower to respond to a mildly threatening stimuli, supporting this “cognitive freezing” effect as a subtle indicator of animal emotion [52]. The ‘moods’ of baboons (*Papio papio*) were measured through spontaneous behavioral indicators where the negative states caused reduced performance in a voluntary cognitive task [57]. Interestingly, the increased feasibility of the response-slowing approach has already resulted in engagement by zoos: the response-slowing of three primate species at Lincoln Park Zoo (Chicago, IL, USA) was tested in relation to anthropogenic noise (jet planes flying overhead), where it was found that Japanese macaques (*Macaca fuscata*) seemed to be disturbed by the noise while the gorillas and chimpanzees were not [53].

## 5. Why Should Zoos Conduct Cognitive Bias Research?

The above studies have successfully tested cognitive bias in many different non-domesticated species, and in the last couple of years a handful of functional zoos have started to conduct these studies as well. In order to understand current and future trends of this specific research in zoos, this review will now investigate why it should be promoted in this setting: what are the benefits to the scientific, management, public and animal stakeholders?

### 5.1. Cognitive Bias Tests Can Inform Us About the Animals’ Welfare

Animal welfare is an all-encompassing concept and notoriously hard to measure, where the most accurate approach is to collect behavioral, physiological and cognitive data over a period of time and then weight the results to achieve some sort of meaningful overall picture of welfare [9,11,67,68]. Although modern zoos report that good animal welfare is a priority and central to their purpose [69], they often do not have the time or resources to measure welfare continuously and scientifically [70,71], bearing in mind that this is still a relatively new branch of science [9]. The application of cognitive bias testing to animals has been the one of the most significant discoveries in the last decade for animal welfare science due to its feasibility and success in reflecting affective states [36,72]. Although cognitive bias testing is not an immediately measurable welfare indicator, it can be used to identify other more practical species-specific measures, which can then be applied more frequently in in situ assessments. As discussed earlier in this review, cognitive bias studies have already revealed key animal-based and resource-based welfare indicators for non-domesticated species often kept in zoos, such as enrichment provision for rhesus macaques [51], certain stereotypic behaviors in tufted capuchins [60], space availability for collared peccaries [59], and synchronous swimming in bottlenose dolphins [14]. Access to group- and even individually-specific welfare indicators such as these would allow zoos to measure welfare more accurately, regularly and therefore optimize the quality of life of the animals in their care [21]. Notably, cognitive bias studies allow the identification of positive emotions and welfare, a far more difficult task than measuring negative states [1]. Such bias tests could be incorporated into overall welfare assessments used by the zoo, or conducted separately as a way to monitor the animals’ mood [73]. Results from cognitive bias studies can also answer specific questions on management and environment changes, and such data on the associated welfare outcomes would be invaluable to regulatory and inspection bodies. Due to the large number of species kept in each zoo, inspectors often do not have species-specific welfare knowledge or the time for in-depth enquiries and would benefit from such objective information [74].

Additional stakeholders invested in the animals’ welfare are the zoo visitors, who are more informed and concerned about the topic than ever before [17,75,76]. Conducting cognitive bias studies in zoos and making the process and results clear to the public, would provide an objective tool for visitors to make their own decisions about the animals’ welfare. Public demonstrations of research protocols have been found to increase zoos visitor knowledge and engagement [77,78]. Since cognitive biases, primarily optimistic and pessimistic traits, are present in humans and easily relatable, this area of research in zoos could be an important bridge linking objective welfare information to visitors and other stakeholders.

### 5.2. Cognitive Bias Tasks Themselves Can be Enriching for Animals

Zoo animals do not face the survival pressures of their wild counterparts and therefore one of main challenges of the captive environment is providing the animals with adequate stimulation [20,70]. Conducting research tasks in zoos can be a significant source of stimulation for the animals, as is often very complementary to existing enrichment programs [20,79,80]. Traditional enrichment often manifests as the addition of structures or objects which aim to stimulate different senses, management of social groupings, visitor interactions, or through training sessions [81,82]. Training in the zoo setting generally describes operant conditioning tasks that use reinforcement (rewards) and/or punishment to increase desired behaviors and reduce inappropriate ones [83,84]. Recently it has been shown that Positive Reinforcement Training (PRT), where only reinforcers are used to condition animals and any incorrect responses are ignored as opposed to punished, can be a significant tool in increasing the welfare of zoo animals (for example, [85,86,87,88,89]). PRT directly increases learning opportunities, human-animal interactions, and can facilitate the provision of other environmental enrichment [85], and therefore when cognitive bias tasks are taught to subjects using PRT techniques, the same benefits will most likely occur [90]. Evidence that animals find cognitive bias enriching is already available: grizzly bears performed anticipatory behaviors before testing sessions [55], previously fearful mink (*Neovison vison*) became more exploratory following the test training [91], and bottlenose dolphins showed positive signs of high arousal (vocalizations, high swim speeds, not leaving test area; I. Clegg, unpublished data) during a judgement bias task [14]. In perhaps the strongest evidential circumstance, two studies tested chimpanzees’ and baboons’ cognitive bias entirely on a voluntary basis where the animals had to approach the experimental area in their enclosure and choose to participate in order for results to be collected [36,57].

The training process is not the only element of cognitive bias studies likely to stimulate the animals: when implementing different emotion-inducing conditions animals may experience new objects or stimuli, access different enclosures, or interact with different conspecifics than in their normal routine. Increased time spent interacting with the humans conducting the tests also has the potential to be enriching and positive Human-Animal Interactions (HAIs) are correlated with better welfare in zoo animals [17,92,93]. Conversely, cognitive bias tasks evidently also have the potential to cause negative states, for example frustration may occur as a result of the non-reinforcement of ambiguous stimuli [94]. Welfare may also be intentionally made poorer, such as in those studies testing the effect of psychopharmaceuticals [95], removal of enrichment [64], or anthropogenic noise [53]. However, these effects are almost entirely dictated by test design, and prudent choices with much preparation can ensure that only positive or neutral welfare consequences are acceptable outcomes, as is likely to be the case in zoo environments.

### 5.3. Findings Can Improve Management of Animals

It was discussed earlier how cognitive bias studies could help to measure and promote good welfare, but this research can also be used more directly to improve the care of zoo animals and promote evidence-based management practices, which should be the goal of any modern zoo [96]. Cognitive bias experiments generally correlate controlled changes to some aspect of the environment with variation seen in biases [1]. These findings therefore demonstrate to caretakers the direct effect of changing the environment or management on the animals and could be actively used by zoos to make a priori predictions and establish different management protocols. This is especially relevant to those understudied or ‘exotic’ species, where little information is available on how to manage them [21,97]. Ensuring appropriate social groupings is one of the primary challenges for managing zoo animals, especially those with complex social networks [98,99,100], and cognitive bias studies have started to reveal the significance of social interactions for some species. Canaries (*Serinus canaria*) were found to make more optimistic judgements when pair-housed as opposed to living solitarily [56]. Higher synchronous swimming was shown to be correlated with optimistic biases in bottlenose dolphins, suggesting a positive affective value of having better social bonds: in turn, this enabled the management team to monitor and manage the social situation more effectively [14]. Cognitive bias studies can also improve understanding of the affective value of hierarchical positions, and thus management: more dominant rats (*Rattus norvegicus*) were shown to make more optimistic judgements [101], as were tufted capuchins [61], and although only tested against hierarchy evaluations made in the past, chimpanzee optimism was significantly correlated with a higher social rank [36]. In terms of stressful management procedures (e.g., veterinary exams [51,52]; and net trapping [58]), cognitive bias testing can help us understand the consequences for the animals, therefore better informing zoo managers and in addition having the potential to improve wild animal management procedures.

Cognitive bias research can also help caretakers to provide the animals with those resources they seem to value, for example enrichment items [15,16,51] or positive human interactions [24]. A recent study with hens (*Gallus gallus domesticus*) showed that increased environmental complexity buffers against the negative effects of stress, as reflected in the animals’ cognitive biases. A recent cognitive bias study on collared peccaries also found environmental enrichment to mitigate the effects of stress [59], and so there is clearly much potential in using cognitive bias to ask pertinent questions about zoo animals’ perception of their environment [25]. Lastly, cognitive bias studies can also highlight how the animals perceive environmental characteristics that may be harder to control but whose effects could be mitigated, such as anthropogenic noise in the zoo [53] or visitor density [16].

### 5.4. Cognitive Bias Research in Zoos Has Intrinsic Value

We can also consider the intrinsic value to science of conducting cognitive bias studies on zoo-housed species, as such research would greatly further our knowledge of animal emotion, learning, and decision-making. There are a large number and variety of species kept in zoos, the majority of which are understudied in terms of their basic biology let alone cognitive and emotional capabilities [96,97]. Cognitive bias tests can support other fields of interest to zoo researchers, for example providing insights on how the animals perceive their environment to complement studies on physiology, ontogeny, kinship and genetics [102]. Animal cognition studies are classically limited by “black box” problems where it is hard to prove what processes and pathways are being used [103], and especially regarding species with higher cognitive capabilities, of which many are often kept in zoos. While they will not answer all our questions about the animal mind, cognitive bias tests on zoo animals would certainly shed some light on the presence and interaction between animal emotions and cognitive processing, and in species with different evolutionary histories. There are still many unknowns and discrepancies with cognitive bias testing itself [5,13,91], and designing and conducting the tests on different species in semi-controlled environments would help to improve the external validity of the approach. The reliability of cognitive bias results could be investigated in zoo settings, especially in relation to persistent, longer term variables such as personality for example, where the life history and longer life spans of zoo animals can be an advantage for repeated testing. The precision of cognitive bias testing could also be tested to a certain extent using species families such as dolphins and great apes in zoos, which tend to participate in advanced training programs [53,104]. These would be ideal animal models for more complex cognitive bias investigations, where the tasks can be meticulously applied and results compared to many other welfare indicators to understand how accurate and responsive bias tests can be.

## 6. Recommendations for Cognitive Bias Tests in Zoo Settings

### 6.1. Which Animals, and What Type of Test?

While conducting cognitive bias tests on many of the species kept in zoos would no doubt be useful to managers and valuable to scientific knowledge, it should be remembered that these are only the first tests in this setting and so should be set up for success, where species choice and test designs could be based on past, similar studies where possible. In this way, these initial tests can be considered as habituating the scientists and zookeepers/managers to the process, paving the way for future similar research, and thus working towards the long-term goal of including many different species.

Before even choosing the species to conduct tests on, it is perhaps more important to understand that the several types of cognitive bias have different requirements for testing, some which might be more conducive to certain species than others. Judgement bias tasks, where the subject must make an active choice in response to a cue, involve a certain level of training of the animals. The training phases are generally organized as: habituation to the cues and apparatus; learning the conditioned cues; probe trials with ambiguous cues [14,37]. Awareness of the benefits of training zoo animals has increased exponentially in recent years and at the same time Positive Reinforcement Training (PRT), previously only used in captive marine mammal facilities, is increasingly the chosen training method [85,87]. Past judgement bias studies on laboratory and farm animals tended to use the operant conditioning approach with a punishment as the outcome of the negative cue, ranging from mild (e.g., waving a plastic bag [105]) to severe (e.g., electric shock [106]). It seems that investigators are slowly moving away from punishment and instead using a combination of positive and negative reinforcement, where food is withheld as an outcome of responding to the negative cue [13]. Very recently judgement bias has been successfully tested using PRT techniques only, i.e., when varying positive outcomes are used for both conditioned cues, and notably these studies have been on zoo species/those also kept in zoos [14,55,60]. While PRT is the method least likely to induce frustration and influence response rates as a result [13], the type of reinforcement used should always be considered in relation to the research questions: for example, using only positive reinforcers may weaken the parallels that can be made with the anxiety-depression model. This human paradigm suggests that depression is linked to a decreased tendency to expect positive events, while anxiety is more closely associated with increased expectation of negative events [1]. Judgement bias tests using punishment outcomes have signaled its presence in animals, but further investigations would have to confirm whether results from tests that do not use “negative events” are still relevant to this specific model. Future studies could also explore whether non-food reinforcers could also be used to test judgement bias, as this might increase feasibility in zoo species that are (seasonally) less food-motivated.

All things considered, given the parallel benefits of PRT training on animal welfare and its increasing adoption in zoo settings [86,87,89], this approach would be a good choice for conditioning to cues in judgement bias tasks. Therefore, when choosing subject species for judgement bias tests, those that already have some experience of training (ideally PRT), or can undergo a gradual training program before the tests begin, would be the best candidates. For untrained animals it is very important to slowly introduce training into their routines before testing cognitive bias because there is some evidence that starting training might in itself be so enriching for the animal that it confounds the bias results [91]. Like judgement bias, testing memory biases and response-slowing also involves an adequate level of training so the same recommendations would apply.

Measuring attention bias requires much less training [1,45,49] and would be especially appropriate for untrained zoo animals, those with shorter attention spans, or those that are managed more extensively. The key to attention bias studies is selecting salient cues which enable reliable testing of the animal’s attention, and if this is achieved, the tests could be conducted in under one minute per animal [49]. It is also likely that some method of moving the animals to an experimental area will be required (which does not induce stress) to control for other environmental stimuli. However, an astute experimental design could potentially allow testing without moving the animals: this would also be a great step forward regarding initiating cognitive bias studies in the wild, something which might not be so far-fetched [66,107] and would help solve “black box” problems associated with studying wild animal cognition [103,108].

Once the requirements for testing the various types of cognitive bias have been understood, the study species should be chosen. Cognitive bias has been successfully measured in a range of vertebrate and a few invertebrate species [109]), although not yet in fish, amphibians, or reptiles [13,21], and so selecting a species need not be limited by position on the phylogenetic tree. If the study and test is carefully planned, cognitive bias should be able to be measured in any species [21], although of course phylogeny will play a part in the study design. Species that have engendered recurring questions regarding welfare in the zoo setting could be prioritized for the first cognitive bias tests, including elephants [6], polar bears (*Ursus maritimus*) [110] and killer whales (*Orcinus orca*) [111].

**Figure 1:**
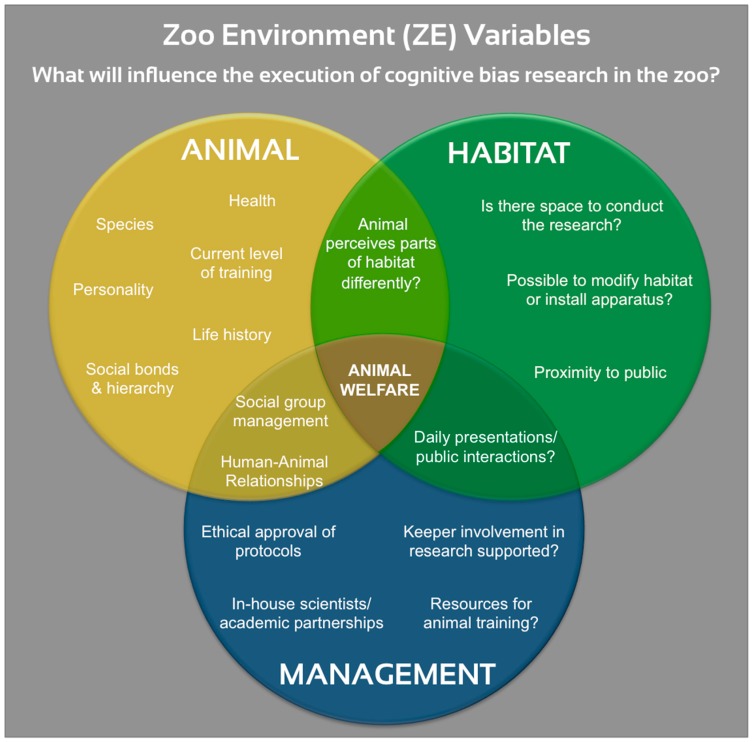
Venn Diagram showing the principal factors to influence cognitive bias studies in zoos, labelled the Zoo Environment (ZE) Variables.

There are many elements specific to the zoo setting which will interact to influence the design and implementation of a cognitive bias study. Given that these elements will be discussed numerous times in following recommendation section, the principal factors are collated here and are labelled the Zoo Environment Variables, or ZE Variables (Figure 1). The diagram is organized into animal, habitat, and management categories, where the central variable that links them all is animal welfare. The welfare state of a zoo animal varies according to management protocols, the habitat it is in, and on a within-individual basis as well. The current balance of affective states, i.e., welfare, of the subject likely has the largest potential to impact the success of cognitive bias tests [36,38], where it can affect the training process, experimental manipulations, and yielded results.

### 6.2. Study Goals: Feasible for Animals, Keepers and Scientists

The likely reason that cognitive bias studies in zoos have been slower to develop is that it requires the synergistic alliance of scientists, zookeepers, management teams and the animals themselves, all taking place within a commercial organization that is open to the public. Cognitive bias studies in zoos are subject to the ZE variables described above (Figure 1), whereas in other settings many of these can be eliminated or controlled. To establish such a unique collaboration, the study’s goals must prioritize mutual benefits and feasibility for each of the stakeholders.

#### 6.2.1. Ask a Research Question Relevant to Subject Animals

When the benefits of cognitive bias testing in zoos were discussed earlier, the majority were centered on improving and optimizing the quality of life of the animals. Therefore, when deciding what research question to ask, i.e., “Is the subject’s cognitive bias affected by X?”, those ZE Variables within the “Animal” category must be considered in relation to the zoo’s management protocols or the animal’s environment (Figure 1). For example, for a species that is generally human-shy in captivity and where the group is managed extensively, a pertinent question might be whether they show increased negative biases during days/periods when keepers or maintenance staff enter the enclosure. Individual differences such as personality and social hierarchy should be taken into account when imposing experimental manipulations to alter welfare state: if the effect of enrichment was being tested, experimenters should be aware that individuals with different personalities might perceive the resource in different ways, and that access to the enrichment itself might be dictated by position in the social hierarchy [15,112,113].

While manipulating zoo animals’ environment can help us answer specific questions on how to manage them, we can also investigate the animals’ spontaneous moods generated from their current environment. Animal ‘moods’ can be conceptualized as the accumulation and interaction of short-term (discrete) emotions, stimulated by their environment, and longer-term affective states which may or may not be derived from an object or event [8]. As with humans, these moods occur spontaneously during the animals’ day-to-day existence and can impact longer-term welfare states, and fortunately cognitive bias tests can give us some direction towards measuring them [8,114]. This approach therefore does not involve manipulating the animals’ environment but aims to correlate cognitive bias with parameters likely related to these mood states. Notably, the few studies employing this approach were mostly conducted with zoo species, and a common result in those contexts was that social bonding and hierarchies were likely strongly influencing the animals’ emotional states [14,36,57]. This type of cognitive bias study is meaningful to the animals as it applies to their everyday environment, as opposed to an imposed situation [14], and would be well-suited to zoo species where interventions are harder to apply due to environmental or ethical limitations.

#### 6.2.2. Thoroughly Involve Animal Caretakers at All Stages

It is generally accepted that zookeepers know their animals best but this has also been proven empirically: caretakers can accurately evaluate the behavior [115,116], personality [117,118] and welfare [115,119,120] of their charges, something that scientific approaches do not always achieve [121]. When initiating a cognitive bias study in a zoo, the caretakers should be involved in the first discussions of the selecting the species and type of test, through to the test design, training stages and interpretation of results. They are rarely formally involved in scientific research, but can provide key information on the normal habits of the animals, their life history, the social hierarchy and interactions, personality, and training capabilities [115,122]. Caretakers also often have unique Human-Animal Relationships (HARs) with their charges which have been shown to enhance welfare [92,123,124]. A good HAR is likely to ensure success in cognitive bias tasks, but also may need to be considered in terms of confounding effects on the bias results. One of the main roles of caretakers in cognitive bias studies is likely to be conducting the training and testing phases. Depending on the ZE Variables (Figure 1), the test can be designed with either direct or protected contact, or with more remote involvement of the keepers. The study’s principal investigators must therefore work very closely with caretakers to ensure the training is conducted accurately, and both groups may need to receive extra instruction in the principles and practice of animal training. A strong and mutually respectful partnership between caretakers and zoo managers and scientists is therefore needed to succeed in testing cognitive bias.

#### 6.2.3. Collaborate with Academic Institutions

In the past decade there have been many calls for scientists to collaborate with zoos, leading to longer-term and formal associations between zoos and academic institutions [19,125,126]. Such partnerships can achieve common research goals, significantly expanding the potential for multi-species studies and providing zoos with empirical information about their animals, most of which can be fed back to improve management. Given the time-investment of cognitive bias testing, and their novelty within zoos settings, it would be beneficial for the zoos to partner with an academic institution containing researchers with expertise in animal cognition or welfare. Zoos with in-house research departments could nevertheless contact such universities to seek advice and guidance through the process, if not a full collaboration. Again, mutual respect and common goals need to be established when partnering, and caretakers and managers from the zoo may have to aid in habituating the researchers to the study species if it is one they are not accustomed to. Academic institutions can provide substantial support when it comes to entering and analyzing the data, which is not always straight-forward with cognitive bias results [22], and publishing the findings.

### 6.3. Designing the Task to Measure Cognitive Bias

Designing or adapting the cognitive bias task itself should involve much research and many iterations, conducting pilot tests where possible to test feasibility. Several reviews have helpfully tabulated existing cognitive bias tests and categorized the different elements, and should form the basis for initial literature reviews to find an appropriate task paradigm [1,13,21]. Of note is a review by Bethell et al. where the authors produced a step-by-step theoretical guide for designing bias tests for fish, amphibians and reptiles [21]. Below, test elements are discussed in relation to application in the general zoo setting, where all judgement, attention, memory, and other bias tasks are considered together unless one type is specified.

#### 6.3.1. Appropriate Cue Choice

Cognitive bias tasks are based on the principle that after using either classical or operant conditioning, an animal will respond in different ways to two cues. The ‘bias’ is then quantified as the response to ambiguous cues: those located at intermediate points between the two conditioned cues on a certain dimension. The choice of cues, and moreover the dimension they are placed on, is therefore key to the validity of the test [13,21]. The majority of past judgement studies have used spatial, visual or auditory cues, and experimenters tend to choose the most biologically relevant to the species [21]. When selecting cues for a study in the zoo setting, previous comparable studies can be consulted (Table 1), but most importantly the ZE Variables will again need to be considered (Figure 1). For example: at the species level, what are the primary sensory modalities for this species, and at the individual level, is it certain that the animal is proficient in using these senses? In the zoo there is less scope to choose the subjects, and therefore it must be verified that the individuals have not suffered a sensory loss or impairment. If spatial cues were chosen for a large terrestrial animal, will there be enough space in their enclosure to establish the cue positions? And if the test area borders close to visitor viewing areas, might the animal be distracted during the task? Cue selection should follow extensive reviews of previous studies, caretaker and management discussions, and pilot tests.

Most cues used in cognitive bias studies lie on the same dimension and therefore the conditioned and ambiguous cues are easy to scale accurately, i.e., auditory cues are often graded frequencies of one sound. Some studies have used multiple dimensions for cues, such as an experiment where different auditory stimuli were chosen (e.g., a whistle, horn, and bells as the intermediates [58]). However, interpreting results and predicting responses is much more difficult when using this method, and the risk of peak shift occurs [13]. The Ambiguous-Cue Paradigm (ACP) does not use a stimulus dimension or continuum but instead pairs ambiguous cues with novel stimuli: although a valid approach that was applied in a zoo setting and deserves future research, the authors were not able to measure cognitive bias in the chosen species [15,16]. It is, therefore, recommended that those looking to conduct their first zoo cognitive bias studies choose cues along a single dimension [13].

The recent focus in attention bias tests has been to eliminate the need for training, and studies have successfully used naturally aversive cues such as predator eyespots [66] or the presence of a dog [49], where no prior conditioning was necessary. If such reliable cues can be identified for zoo species this would be a viable and practical method to use, but a limitation could be that ethical questions might arise from potentially inducing fear responses in the animals.

#### 6.3.2. Test Procedure

Judgement bias test procedures in particular can vary significantly in terms of choice of reinforcement/punishment, number of ambiguous cues, Go/No-Go or Go/Go (active choice) tasks and number of trials: discussions on these aspects can be found in multiple other reviews [12,13,21]. In relation to the zoo setting, a few recommendations are outlined here which might aid in defining test procedures. Firstly, regarding reinforcement during the task: since PRT has been shown to be conducive to better welfare and management in the zoo [86,89], and successful in testing cognitive bias [14,55,61], it should be chosen where possible. If using PRT, pre-experimental testing is often needed to validate the of the value of different reinforcement for the conditioned cues which can then be considered the ‘positive’ and “less-positive” cues [55,89]. A secondary reinforcer, i.e., a signal to let the animal know it has performed the correct behavior, can be used in cognitive bias tasks and may be beneficial in maintaining responsiveness and avoiding frustration [13]. If the animals are not learning the difference between these conditioned cues there is always the possibility of making the difference more extreme. In these cases, and for studies expressly wishing to investigate the anxiety-depression model in animals [1,35], the outcome for the ‘less-positive’ cue might need to be the withholding of the reward (i.e., negative reinforcement) [21].

During Go/No-Go tasks for judgement bias, the positive cue elicits an active response and the negative/less-positive cue results in no response. The other main type used has been Go/Go paradigms, where two different but active responses are required after both conditioned cues. Go/No-Go tasks may be slightly easier to train and establish, but Go/Go tasks have been increasingly used in the last few years as they control for the different activity levels between subjects [13,21]. In both approaches, past investigators have either partially or never reinforced the ambiguous cues, which has split opinions regarding which would ensure validity with the least influence on future trials. Again, with these decisions the ZE Variables should help to inform choices, in particular the individual differences and level of training (Figure 1) since the tasks and reinforcement schedule must ensure the animals stay motivated to participate. An interesting recent improvement was proposed for Go/No-Go tasks, where subjects could initiate the next trial after No-Go cues in an effort to prevent frustration and add control for the animals [94]. Bethell et al. provide a useful decision tree schematic for choosing tasks, cues and reinforcement for frogs, amphibians and reptiles [21].

#### 6.3.3. Social Isolation: Not Always Necessary

The perceived need to socially separate animal subjects for non-invasive behavior or cognition testing is partially linked to the traditional laboratory research ideology where sterile, fully controllable and isolated environments were the norm for such tasks and observations [127,128]. However, we now know that creating such environments can skew results and significantly decrease accuracy and validity [129]; the same has been concluded for cognitive bias experiments on zoo species which has led to some studies testing the animals without socially isolating them [14,57,58] (Table 1). This has been achieved through: training the cue discriminations to the animals as a group [58]; engaging the rest of the social group in separate training sessions [14]; or using a touchscreen and establishing voluntary participation [57]. In one study, the actual solitary isolation (and subsequent test participation) was achieved voluntarily [36] and thus also negated the potentially confounding effects of isolation stress on the animals [13,130]. If isolation is necessary, it may be less stressful to temporarily separate them in a section of their home environment than move them to an unfamiliar testing arena, although it should be considered what established values the animals have for different parts of their home enclosures (e.g., areas close to where they are usually fed). The impact of separating animals varies greatly between species, context and individuals [131,132], so in some cases it might be entirely appropriate to isolate for testing, but the animals must of course still be habituated to being separated and to the isolation area [133,134]. For social species, investigators should consider maintaining visual and auditory contact between the animals to decrease stress levels [35,56]. Well-trained “gating” behaviors, or the ability to reliably position the animals in different parts of their enclosure, will be an advantage for those aiming to test cognitive bias within social groups as well as for isolating individuals.

#### 6.3.4. Interpretations and Limitations of Cognitive Bias Data

Once the tests have been designed, conducted, and the data collected, care must also be taken over the analysis and interpretation of results. Although dozens of cognitive bias studies have been carried out and have successfully reflected changes in emotional state, the results have been analyzed in a surprising variety of ways. Chiefly, the final measure of the bias itself has previously been calculated from the responses to one, some, or all of the ambiguous cues, and has either been anchored to the conditioned cues or considered separately [22]. A recent review of judgement bias studies aimed to develop a coherent statistical approach for such tests, and recommended that analyses are best conducted using mixed effects models that accounts for the behavioral variable used to measure bias, any dependencies in the data as random effects, and incorporates responses for each trial within the outcome variable [22] (pp. 65–66). While not all those working on cognitive bias agree with these recommendations [135], greater cohesion in analytical methods would facilitate comparisons between methods and species and allowing the field to advance more effectively [13]. Making data publicly available through repositories would also enable more between-study comparisons and meta-analyses.

We must also be cognizant of the limitations of cognitive bias studies, some of which might be more pronounced in the zoo setting. Firstly, the collective experience of testing cognitive bias is still relatively new and despite most results agreeing with a priori hypotheses, there are some that hint at many further complexities within this phenomenon. For example, rescued goats (*Capra hircus*) that had previously been neglected displayed optimistic biases when tested, despite their current good environment [136]. This suggests that in addition to reflecting the opportunities and threats in the immediate environment, cognitive bias may also depend on an animal’s past environment many years ago. Another significant unknown is the degree to which cognitive biases may be phenotypic traits, and not just transient states. It is well-accepted that a certain level of optimism or pessimism can be a stable trait in humans, and although a few studies have found the same in animals, the focus has remained strongly on cognitive bias as a measure of shorter-term welfare and mood [137,138]. Further testing on the repeatability of cognitive bias results would therefore be useful, as well as the impact of within-test repeatability given that animals’ response to the ambiguous cues is likely to change no matter how they are rewarded. Therefore, given these on-going areas of investigation, we should be cautious when interpreting results and willing to consider and discuss alternative explanations. The cognitive bias results of one animal or group should not be extrapolated to others without reassessment, and regular monitoring of an animal’s bias should be encouraged where possible. Regarding the practical applications of cognitive bias testing for welfare assessments, we should also be aware that although it is one of the most valid approaches for measuring longer term emotional states currently available, it should still be used in conjunction with other measures [139], as this will help to ensure the construct validity of the tests. Only welfare evaluations that employ a suite of multidisciplinary indicators can hope to make a reasonable estimation of the animals’ actual welfare state [9,140].

## 7. Conclusions

Cognitive bias testing is a valuable tool in understanding the effect of an animals’ environment on its affective state and has been conducted with several species in many different settings. Judgement bias tasks have been the most common type of test applied, but recently improvements regarding feasibility and training time have resulted in the development of new paradigms. While only a few cognitive bias studies have taken place in the zoo environment, there have been many on “non-domesticated” or “exotic” species kept in other institutions, and these can be drawn upon when designing the first tests within functional zoos. There are many reasons why zoos should conduct cognitive bias studies: they can provide valuable information on the animals’ welfare, which can then inform management protocols, but the testing can also be enriching in itself for the animals and the results have an intrinsic value for the advancement of our knowledge of animal emotion and cognition. To design a cognitive bias study for zoo animals, investigators should carefully consider the context-specific animal, habitat and management factors which will affect the success of the tests. They have been categorized and named here as the Zoo Environment Variables (ZE Variables) and are generally applicable for the design of any type of research study in the zoo setting. The ZE Variables will influence or even dictate many of the elements in cognitive bias testing, and one of this review’s major recommendations is that zookeepers and scientists closely collaborate at each stage of the process to ensure the study’s feasibility and validity. Positive Reinforcement Training will likely be a key tool in the success of judgement bias studies, although more extensively managed species can still be studied using other paradigms, and social isolation is not always necessary. Cognitive bias studies undoubtedly involve meticulous planning and interdisciplinary collaboration, and this is even more true in zoo settings, but it is hoped that this review’s recommendations will guide and encourage the process. Cognitive bias tests are certainly feasible in zoos and a few pioneering studies have already showcased the many benefits to managers, scientists, and the welfare of the animals themselves.

## Figures and Tables

**Table 1:** Published cognitive bias studies conducted in zoo environments, and those carried out in other settings but involving non-domesticated, “exotic” species often kept in zoos; adapted from [13]. D: was the animal’s affective state altered experimentally as part of the design, or were bias results correlated with spontaneous mood states? E: what type of task and cues were used? F: was negative reinforcement/punishment used for differentiating the conditioned cues (S+/S−), or was positive reinforcement used (S++/S+)? G: were the subjects socially isolated, and tested in their home environment? H: was the null hypothesis (H_0_) rejected, i.e., was cognitive bias significantly linked to affective state indicators?

A	B	C	D	E	F	G	H	I
Species	Setting	Type of Cognitive Bias	Experimental Manipulation?	Task and Cues	S+/S− or S++/S+?	Tested in Isolation or Group; in Home Environment?	Link between Cognitive Bias and Affective State?	Reference
Common marmosets, *Callithrix jacchus*	Laboratory	Judgement bias	Yes: rearing context. Either: family-reared twins, family-reared animals from triplet litters where only two remain, or supplementary fed triplets.	Go/No-Go; visual cues: height of wooden tubes	S+/S−	Isolated; home	No: overall no consistent effects of rearing context	Ash and Buchanan-Smith 2016 [50]
Chimpanzees, *Pan troglodytes*	Rescue center	Judgement bias	No: individual differences	Go/No-Go; visual cues: color of paper cone	S+/S−	Isolated; home	Yes, correlation with rank, higher rank = less pessimistic	Bateson and Nettle 2015 [36]
Rhesus macaques. *Macaca mulatta*	Laboratory	Judgement bias	Yes: environmental enrichment vs. veterinary examination	Go/No-Go; visual cues: length of line on screen	S+/S−	Isolated; home	Yes: enrichment and no veterinary examination decreased pessimism	Bethell et al. 2012 [51]
Rhesus macaques. *Macaca mulatta*	Laboratory	Attention bias	Yes: environmental enrichment vs. veterinary examination	Vigilance towards aggressive images of conspecific faces measured	n/a	Isolated; not in home	Yes: veterinary examination caused avoidance of images, enrichment caused sustained vigilance	Bethell et al. 2012 [45]
Rhesus macaques. *Macaca mulatta*	Laboratory	Response- slowing	Yes: some animals underwent a veterinary examination	Speed to touch 2D shape on touchscreen next to different images of conspecific faces	n/a	Isolated; home	Yes: undergoing a veterinary examination caused slower responses when negative emotional content (images of staring conspecific faces) present	Bethell et al. 2016 [52]
Bottlenose dolphins, *Tursiops truncatus*	Zoo	Judgement bias	No: measured spontaneous social and anticipatory behaviors	Go/Go; spatial cues: position of a target	S++/S+	Group; home	Yes: increased synchronous swimming and decreased anticipatory behavior correlated with more optimistic responses	Clegg et al. 2017 [14]; Clegg and Delfour 2018 [42]
Chimpanzees, *Pan troglodytes*	Zoo	Response- slowing	No: used spontaneous anthropogenic overhead noise from annual aircraft show	Speed to touch 2D shape on touchscreen next to different images of conspecific faces	n/a	Group; home	No: loud sound event did not seem to impact affective state	Cronin et al. 2018 [53]
Gorillas, *Gorilla gorilla gorilla*	Zoo	Response- slowing	No: used spontaneous anthropogenic overhead noise from annual aircraft show	Speed to touch 2D shape on touchscreen next to different images of conspecific faces	n/a	Group; home	No: loud sound event did not seem to impact affective state	Cronin et al. 2018 [53]
Japanese macaques, *Macaca fuscata*	Zoo	Response- slowing	No: used spontaneous anthropogenic overhead noise from annual aircraft show	Speed to touch 2D shape on touchscreen next to different images of conspecific faces	n/a	Group; home	Yes: anthropogenic noise caused slower responses when negative emotional content (images of staring conspecific faces) present	Cronin et al. 2018 [53]
Orange-winged parrot, *Amazona amazonica*	Laboratory	Attention bias	No: personality assessment using subjective ratings	Performance on a foraging task with and without an unfamiliar observer	n/a	Isolated; home	Yes: more neurotic parrots showed greater attention bias, i.e., performed worse in task when unfamiliar human present	Cussen and Mench 2014 [40]
Common marmosets, *Callithrix jacchus*	Laboratory	Judgement bias	No: handedness of animals, data taken from retrospective records	Go/No-Go; visual cues: color of lid	S+/S−	Isolated; home	Yes: left-handed marmosets were more pessimistic	Gordon and Rogers 2015 [54]
Grizzly bears, *Ursus arctos horribilis*	Research, Education and Conservation center	Judgement bias	Yes: environmental enrichment given	Go/Go; visual cues: color of boards	S++/S+	Isolated; home	No: environmental enrichment did not seem to alter affective state	Keen et al. 2014 [55]
Domestic canaries, *Serinus canaria*	Laboratory	Judgement bias	Yes: housed singly or in pairs, and personality measured through behavior coding,	Go/No-Go; spatial cues, position of food dishes	S+/S−	Isolated; not in home	Yes: pair-housed canaries judged more optimistically (but personality did not have an effect)	Lalot et al. 2017 [56]
Baboons, *Papio papio*	Laboratory	Response-slowing	No: measured spontaneous positive, neutral, and negative valence social and solitary behaviors	Computerized visual search task	n/a	Group; home	Yes: negatively valenced behaviors slowed following performance in task	Marzouki et al. 2014 [57]
Gorillas, *Gorilla gorilla gorilla*	Zoo	Ambiguous-cue paradigm	Yes: forage given as enrichment	Visual cues: 2D shapes on a touchscreen	S+/S−	Isolated; home	No: environmental enrichment did not seem to alter affective state	McGuire et al. 2017 [15]
American black bear, *Ursus americanus*	Zoo	Ambiguous-cue paradigm	No: measured spontaneous visitor density	Visual cues: 2D shapes on a touchscreen	S+/S−	Isolated; home	No: visitor density did not seem to alter affective state	McGuire et al. 2017 [16]
White-lipped peccaries, *Tayassu pecari*	Laboratory	Judgement bias	Yes: net-trapping or a control	Go/No-Go; auditory cues: different (multi-dimensional) tones	S+/S−	Group training, isolated testing; home	Yes: net-trapping made animals more pessimistic	Nogueira et al. 2015 [58]
Collared peccaries, *Pecari tajacu*	Laboratory	Judgement bias	Yes: space restriction in interaction with environmental enrichment	Go/No-Go; auditory cues: different (multi-dimensional) tones	S+/S−	Isolated; not in home	Yes: space restriction caused more pessimistic judgements, and effects were mitigated by enrichment	Oliveira et al. 2016 [59]
Tufted capuchins, *Cebus apella*	Laboratory	Judgement bias	No: measured stereotypic behaviors	Go/Go: visual cues: length of rectangles on board	S++/S+	Isolated; home	Yes: monkeys performing more stereotypic head twirls judged more pessimistically	Pomerantz et al. 2012 [60]
Tufted capuchins, *Cebus apella*	Laboratory	Judgement bias	No: measured rates of conspecific grooming and hierarchical rank	Go/Go: spatial cues: position of rectangular object	S++/S+	Isolated; home	Yes: more dominant monkeys and those who received more conspecific grooming were more optimistic	Schino et al. 2016 [61]
